# Therapeutic challenge: Unusual coexistence of idiopathic central diabetes insipidus and diabetes mellitus in a male with vitiligo

**DOI:** 10.22088/cjim.12.0.363

**Published:** 2021

**Authors:** Marcio José Concepción-Zavaleta, Diego Martin Moreno Marreros, Eilhart Jorge García Villasante, Esteban Alberto Plasencia-Dueñas, Sofia Ildefonso Najarro, José Carrion Rojas, Carmen Luisa Achahui Acurio

**Affiliations:** 1 Division of Endocrinology. Clínica Stella Maris, Lima, Perú; 2 National University of Trujillo, Faculty of Medicine, Trujillo, Perú; 3 Division of Endocrinology, Hospital Nacional Daniel Alcides Carrión, Lima, Perú; 4 Division of Endocrinology, Hospital Nacional Guillermo Almenara Irigoyen, Lima, Perú; 5 Division of Endocrinology, Hospital Nacional Guillermo Almenara Irigoyen, Lima, Perú

**Keywords:** Central diabetes insipidus, Diabetes mellitus, Water deprivation test, Vitiligo.

## Abstract

**Background:**

Idiopathic central diabetes insipidus (DI) is a rare endocrine disorder that results from total or partial deficiency of vasopressin secretion. It is idiopathic when the cause is unknown, but in many cases, is associated with autoimmune disorders.

**Case presentation:**

We present the case of a 44-year-old male with vitiligo and a family history of diabetes mellitus and thyroid disease. The patient presented with polydipsia and polyuria greater than 8 L/day. After water deprivation test, the patient was diagnosed with partial central diabetes insipidus. Contrast-enhanced pituitary magnetic resonance imaging showed decreased brightness of the neurohypophysis and normal thickness of the pituitary stalk. Because desmopressin was not initially available, the patient was managed with chlorpropamide, carbamazepine, and hydrochlorothiazide, and afterwards substituted. During his outpatient checkups, he presented many episodes of polyuria, the last after 13 years, with polyuria of up to 15 L associated with weight loss, and abnormal blood glucose levels; anti-GAD 65 and IA-2 antibodies were negative. He was subsequently diagnosed with diabetes mellitus and received metformin and insulin; this latter was suspended in subsequent check-ups due to hypoglycemic episodes.

**Conclusion:**

We highlight the importance of treatment and adequate control of these pathologies, since they share similar clinical manifestations, can easily have electrolyte imbalance and represent a challenge for endocrinologists and internists.

Central diabetes insipidus is a rare disease of the hypothalamic-pituitary axis caused by deficiency of vasopressin synthesis in the hypothalamus, or a lack of secretion in the neurohypophysis (1, 2). The prevalence of central diabetes insipidus is 1 case for every 25,000 people, and the majority of cases are acquired, mainly as a result of craniopharyngioma and germ cell tumors (3). Nevertheless, the etiology remains unknown in 15%–50% of patients, in which the disease is classified as idiopathic (2, 3). The idiopathic form of diabetes insipidus is associated with the presence of autoantibodies against ADH-secreting neurons (AVPcAb), which are present in one third of the cases (4). Studies have shown that these autoantibodies are present in the serum of most patients with pituitary diseases (5). This form of diabetes is often accompanied by thyroid, rheumatologic, or dermatologic autoimmune disorders that increase the probability of immune etiology (1). Identification of AVPcAb and advances in imaging techniques have led to fewer reports of idiopathic (6). We present the case of a male patient with vitiligo, who initially developed idiopathic central diabetes insipidus and later diabetes mellitus, highlighting the challenge of treating and controlling both diseases at the same time.

## Case presentation

A 44-year-old male patient diagnosed with vitiligo at 10 years old, came to the emergency department due to excessive thirst and polyuria of 8 to 10 L/day. The patient had a family history (father and 3 siblings) of type 2 diabetes mellitus and autoimmune hypothyroidism. 

He was subsequently hospitalized in the endocrinology service with diagnosis of polydipsia-polyuria syndrome and high suspicion of diabetes insipidus. On physical examination the following were noted: Blood pressure, 110/70 mmHg; heart rate, 65 bpm; respiratory rate, 16 breaths per minute; body weight, 81 kg (178.5 lb); height, 170 cm; and BMI, 28 kg/m2. 

**Figure 1:**
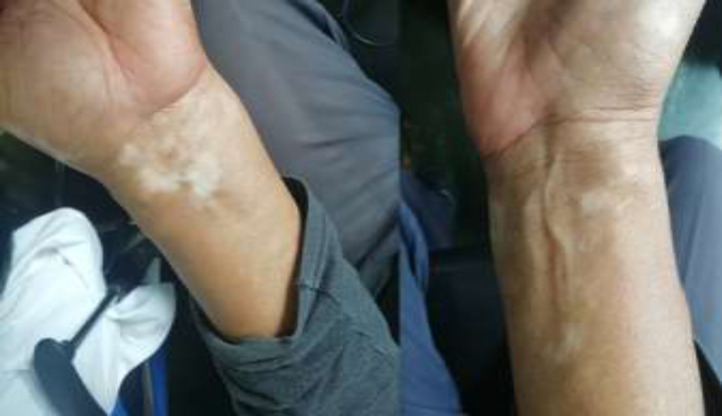
Clinical characteristics of the patient. Hypopigmented macular lesions on both forearms compatible with vitiligo

In the preferential examination multiple irregular hypopigmented maculas were found in different parts of the body, compatible with vitiligo (figure 1).

Also decreased the baresthesia and pallesthesia sensitivity in feet. The remainder of the examination was unremarkable. Biochemical and hormonal examinations revealed the following: serum Na, 141 mEq/l; serum K, 3.84 mEq/l; fasting blood glucose, 110 mg/dl; serum creatinine, 0.81 mg/dl; thyroid profile with free t4, 1.25 µg/dl (normal range: 0.8–1.7 µg/dl); TSH, 2,248 µUI/ml (normal range: 0.4–4 µUI/ml); antimicrosomal antibodies, 32.9 U/ml (normal range: <60 U/ml); and HbA1c at 6.2%. 

Given the presumptive diagnosis of diabetes insipidus, the patient underwent a water deprivation test (Miller test), and after the subcutaneous administration of 5U vasopressin (desmopressin phase), he was subsequently diagnosed with partial central diabetes insipidus (table 1). 

**Table 1:** Results of the water deprivation test (Miller test)

	**0 hours**	**2 hours**	**5 hours**	**2 hours post Administration subcutaneously of 5U of vasopressin**
Body weight (Kg)	82	81	79.4 (↓ 3 %)	78.9
Plasma sodium (mEq/l)	141	144	149	148
Plasma osmolarity (mOsm/Kg)	302	308	318	316
Urinary osmolarity (mOsm/Kg)	235	242	245	281.2 (↑ 14.8 %)
Urine Specific gravity	1.002	1.005	1.007	1.008
Urination Volume (ml)	0	1730	1600	700

**Figure 2:**
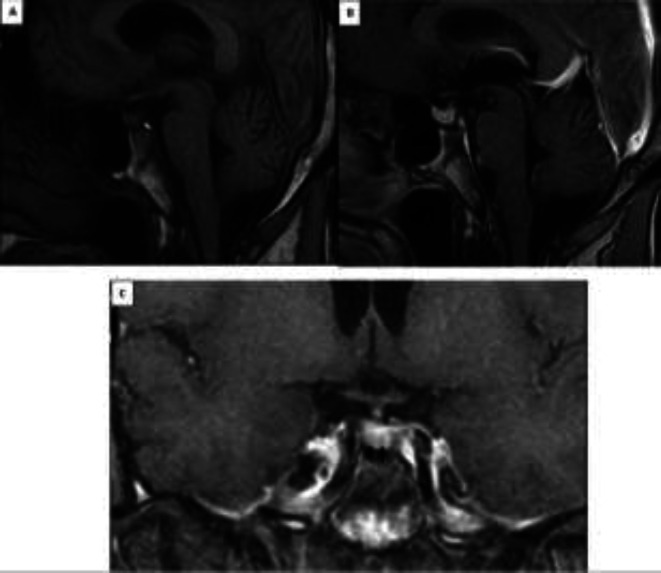
Magnetic resonance imaging of the pituitary gland. A. Sagittal view. Filiform neurohypophysis is observed with decrease intensity (white arrow) in the T1 sequence without contrast. B and C. Sagittal and coronal view. Two microadenomas of 4×2

To identify the etiology, we requested a magnetic resonance imaging (MRI) of the pituitary with gadolinium (figure 2), which revealed the presence of two microadenomas (4×2 mm and 2×3 mm) in the posterolateral region of the adenohypophysis, a filiform neurohypophysis with decreased signal intensity, and normal thickness of the pituitary stalk. Likewise, due to blood glucose and HbA1c values, he was diagnosed with prediabetes.

The patient received initially pharmacological treatment based on chlorpropamide 100 mg QD, carbamazepine 200 mg TID and hydrochlorothiazide 25 mg QD, because desmopressin was not available at that time. In addition, it was recommended to limit the intake of foods rich in sodium and simple sugars, as well as to undertake regular physical activity and maintain an adequate state of hydration. 

The patient had favorable response to the treatment, and was discharged with good tolerance and adherence to the medication. Ophthalmological and auditory evaluation was performed, and showed no abnormalities. However, during the outpatient controls, he had multiple episodes of polyuria requiring hospitalization, that were attributed to the decompensation of the underlying disease, the last being 13 years after diagnosis with polyuria of up to 15 L, which was associated with a weight loss of 10 kg, and strikingly, random blood sugar of 310 mg/dl. This was the last cause of hospitalization for glycemic management.

Because the patient had a family history of autoimmune hypothyroidism, as well as a personal history of vitiligo and idiopathic central diabetes insipidus by probable autoimmune etiology, we suspected a presentation pattern of latent autoimmune diabetes in adults (LADA 2). In line with this, we requested antibodies to glutamic acid decarboxylase 65 (GAD-65) and islet antigen 2 (IA-2) antibody, both of which were negative. 

Consequently, type 2 diabetes mellitus was diagnosed considering that the patient was overweight and had a family history. The results of the blood tests were obtained as follows: HbA1c was 8.2% and basal C-peptide was 0.32 nmol/l.

Glycemia was controlled by administration of metformin 850 mg QD and progressive titration of the insulin dose until a schedule of insulin NPH 20 units in the morning and 10 units at night was established. During hospitalization, the drugs used for the management of diabetes insipidus were replaced by intranasal desmopressin 10 µg at night because this medication was already available in our country, with no adverse reactions. 

One month later during his out-patient control, he reported two episodes of hypoglycemia and a result of fasting blood glucose of 83mg/dl, so we decided to withdraw insulin therapy. The next control in six months revealed serum sodium of 139 mEq/l, fasting blood glucose of 133 mg/dl, and HbA1c of 6.9%. Currently, the patient is asymptomatic and follows his out-patient controls by our service.

## Discussion

Central diabetes insipidus (CDI) is a rare endocrine disorder, the basis of which is the total or partial absence of vasopressin; this is generally due to the destruction-degeneration of magnocellular neurons of the supraoptic and paraventricular nucleus in the hypothalamus, or alterations in their transport from the neurohypophysis to the circulation (1, 2). The typical clinical manifestations are polydipsia and polyuria, which in severe cases can cause serious neurological symptoms. Our patient presented with these typical symptoms, and as a result, a water deprivation test was performed. The laboratory parameters, and the increase of 14.8% in the urinary osmolarity after administering vasopressin, guided us to the diagnosis of partial central diabetes insipidus.

One of the challenges of CDI is to establish the etiology, which can either be of genetic origin due to mutations in the AVP gene locus or acquired. The latter being the most frequent etiology, in which tumors of the central nervous system (e.g., craniopharyngiomas, germinomas), postsurgical, post-traumatic states, and infections represent the main causes (3). It has been reported that the cause of disease for 15%–50% of these patients is unknown and they are included in the idiopathic classification (6). Numerous questions have been raised regarding the cause of idiopathic CDI, mainly highlighting a probable autoimmune origin. Pivonello et al. (12) described the presence of AVPcAb in 32.8% of the 64 patients with idiopathic CDI. The fact that AVPcAbs were recognized in only one third of such patients indicated that they are subject to early disappearance or that autoimmune T-cell local damage may have taken place (15); however, it cannot be ruled out that it may be an epiphenomenon. De Bellis et al. (11) studied the autoimmune cause and radiological characteristics in the pituitary MRI of 22 CDI patients, and reported that 68% of the patients had autoantibodies to AVPcAb in different titers; 7 of whom had thickening of the pituitary stalk. Furthermore, it was evidenced that all the patients presented loss of hyperintense signal of the neurohypophysis correlated to the vasopressin deficit expected of this disease.

The AVPcAb are generally not available in routine laboratories of our country and, if there is, in most cases their use is restricted for research. The consideration of this etiology is most suggestive in the presence of other concomitant autoimmune diseases, such as vitiligo described in this report (3). Patients with autoimmune CDI have an increased incidence of other organ-specific autoimmune endocrine disease, particularly thyroid disease (14). Furthermore, it has been proposed that vitiligo, together with the early onset of the disease and precise radiological changes at the level of the pituitary stalk, could explain the autoimmune origin of this disease with a probability of up to 99% (12). Nevertheless, the presence of AVPcAb has also been found in patients with Langerhans cell histiocytosis or germinomas which indicate that they are not exclusive markers for autoimmune etiology of central diabetes insipidus (11). The patient has a personal history of vitiligo since adolescence and a family history of autoimmune thyroiditis. In addition, the pituitary MRI demonstrated a decrease in the hyperintensity of the neurohypophysis and no thickening of the pituitary stalk, both of which are closely associated with autoimmune pituitary processes (13). However, the diagnostic sensitivity of lack of brightness in the neurohypophysis in cases of partial CDI is <60% (7). The adenomas described were irrelevant in the etiology in this disease, because they were small (microadenomas), non-invasive, and not secreting hormones, in fact non-functioning pituitary adenomas (NFPA). Additionally, data from Europe, North and South America have estimated the prevalence of clinically relevant NFPA to be 7 to 41.3 cases per 100,000 population (18).

There are multiple reports of the association between diabetes mellitus and diabetes insipidus in Wólfram syndrome, a neurodegenerative disease of recessive genetic origin, in which the main characteristics are type 1 diabetes, bilateral optic atrophy, and development of diabetes insipidus (8). However, this diagnosis is unlikely since the patient presented a normal ophthalmological and hearing examination, and the anti-GAD 65 and IA-2 antibodies were negative. Few cases have been reported where both pathologies coexist (9, 10), and in all of these, the diagnosis of diabetes mellitus was made before that of diabetes insipidus. In this way, to our knowledge, this is the first clinical case reported in which diagnosis of diabetes insipidus was made almost 13 years before than diabetes mellitus, and during this time the desmopressin, drug of choice for his treatment, was unavailable in our country.

The patient developed exacerbations in his symptoms that were attributed to irregular treatment and a lack of periodic control of the underlying disease; however, it was postulated that some episodes may have been due to increased blood glucose levels, secondary to lack of control of diabetes mellitus, not yet diagnosed, or by a poor diet control, which generated an increase in plasma osmolarity that could not be compensated by insufficient vasopressin and fluid intake. In addition, the patient used hydrochlorothiazide for more than ten years and it could contribute to the progression from pre-diabetes to diabetes. Zhang et al. established that the use of thiazide diuretics is significantly associated with higher blood glucose levels (16). The exact mechanism of hyperglycemia remains unknown, but it has been proposed that it may be related to increased insulin resistance, inhibition of glucose uptake, and decreased insulin release (17). Therefore, the better control of blood glucose was influenced by the withdrawal of hydrochlorothiazide. On the other hand, chlorpropamide, which is an effective antidiuretic agent for vasopressin-sensitive diabetes insipidus (19), also belongs to the family of first-generation sulfonylureas, so it could initially help to control diabetes mellitus. Chlorpropamide is no longer used for lowering blood glucose (20). Accordingly, the control of both pathologies requires achieving glycemic control and maintaining an adequate hydroelectrolytic and hydration state, for which treatment adherence is required. 

In conclusion, the relevance of this case lies in the management and control of both pathologies together. This remains a medical challenge, since both share similar symptoms, can easily have electrolyte imbalance, and their coexistence is considered to be relatively rare. 
